# Based on the relationship between anxiety of existential meaninglessness, hope level, and fear of progression, explored the effect of preoperative nursing with Orem theory in the senile cataract population

**DOI:** 10.3389/fpsyg.2024.1358229

**Published:** 2024-05-10

**Authors:** Yanli Zhang, Yanmiao Cheng, Yan Liang, Mengfei Shao, Aiai Chen

**Affiliations:** Ningbo Eye Institute, Ningbo Eye Hospital, Wenzhou Medical University, Ningbo, China

**Keywords:** senile cataract, existential meaninglessness anxiety, Herth hope index, fear of progression, relationships, Orem’s theory

## Abstract

**Background:**

Cataracts, characterized by a decrease in vision due to the clouding of the lens, can progress to blindness in advanced stages. The rising incidence of cataract cases has led to a significant number of patients experiencing negative emotions associated with vision loss, thereby diminishing their quality of life. In clinical practice, it is imperative for healthcare professionals to consider the psychological well-being of cataract patients. Currently, there is a scarcity of research focusing on psychological evaluations, such as assessing feelings of meaninglessness among individuals with cataracts.

**Objective:**

This study aims to investigate the factors influencing the anxiety of existential meaninglessness and to explore the relationships among existential anxiety, Herth hope index levels and fear of progression in the elderly cataract-affected population. Additionally, it evaluates the effectiveness of Orem’s nursing care strategies.

**Methods:**

Utilizing a sociodemographic questionnaire, the Existential Meaninglessness Anxiety Scale (EM-A), Herth Hope Index Level Scale, and the Fear of Progression Questionnaire-Short Form (FoP-Q-SF), this research employed convenience sampling for a cross-sectional and intervention study. The retrospective study sample comprised 1,029 individuals, while the intervention study included 317. The intervention design assessed psychological changes in existential meaninglessness following Orem’s preoperative nursing interventions. Multiple linear regression analysis was employed to ascertain the determinants of EM-A within the population of elderly patients with senile cataracts. Pearson correlation analysis elucidated the relationship between EM-A, levels of hope, and the FoP-Q-SF among this demographic. Subsequent investigations, utilizing a *t*-test, evaluated the effects by comparing the data before and after the implementation of the interventions.

**Results:**

The correlation between EM-A, hope levels, and FoP-Q-SF was statistically significant (*p* < 0.05). Factors such as age, education level, alcohol consumption habits, hope levels, and FoP-Q-SF scores significantly affected EM-A scores (*p* < 0.05). Orem’s nursing framework significantly reduced existential anxiety (*p* < 0.05).

**Conclusion:**

Among elderly patients with cataracts, existential anxiety was generally moderate. Hope levels and fear of progression were closely associated with the EM-A. The novel Orem preoperative care model effectively addresses clinical issues. In clinical practice, it is crucial to address psychological problems and enhance patients’ quality of life.

## Introduction

With the advent of an aging society, China has experienced a steady increase in cataract prevalence. From 1990 to 2019, the total number of cataract cases in China surpassed 1.8 million, with projections indicating a continued rise in the cataract burden from 2020 to 2030 (Wu et al., 2023). Cataracts, characterized by degenerative changes in crystalline lens metabolism leading to lens opacity (Delbarre and Froussart-Maille, 2020), primarily affect the middle-aged and elderly populations, with age and diabetes identified as key risk factors (Lim et al., 2020; Dai and Zhu, 2022). Early symptoms often go unnoticed (Nakazawa et al., 2020), and without effective intervention, severe cases can lead to complete blindness (Interlenghi et al., 2023).

Vision is the most critical sensory component of the human information system, and enduring vision problems or loss can significantly impair daily living skills and jeopardize psychological stability (Mylona et al., 2021; Guan et al., 2023). Research indicates that the severity of an ophthalmological diagnosis is directly correlated with increased depression levels, with cataract patients being particularly prone to depression (Pop-Jordanova et al., 2014). Additionally, individuals suffering from both cataracts and diabetes exhibit a higher susceptibility to depression compared to those with only diabetes (López Sánchez et al., 2021). While some studies suggest that male cataract patients are more likely to experience major depressive disorders (MDD) than females (Kang et al., 2023), others argue that females are at a greater risk for developing cataracts (Wu et al., 2023).

Visual impairment also diminishes social engagement among the elderly, further isolating them from society and causing significant psychological distress. Severe cataracts can lead to decreased cognitive functions, limiting social interactions and adversely affecting psychological well-being (Agarkov et al., 2022). In mental health, depression can trigger a cascade of negative effects, including feelings of existential meaninglessness and a reduced hope for life (Binder, 2022). Chronic diseases are associated with an increased sense of existential meaninglessness (Xue et al., 2019). Notably, effective preoperative care can ameliorate the psychological state of patients (Zhang et al., 2023).

Orem’s Self-Care Theory suggests that nurses should interact with patients based on their self-care capabilities: total compensation, partial compensation, and supportive educational systems. In the supportive-educational system, patients are capable of self-care but require education on therapeutic self-care behaviors (Yang and Niu, 2022). Nurses are optimally positioned to identify potential health issues and provide appropriate supportive educational interventions (Dinkelaker, 1999; Orem, 2001; Cottin et al., 2004). Recent research highlights that nursing interventions based on Orem’s theory can enhance patients’ self-efficacy and, consequently, their quality of life and mood (Hashemi et al., 2014; Deng et al., 2021; Mengmeng et al., 2023).

Hence, given the absence of relevant assessments for cataract patients, this study aims to evaluate the factors contributing to their sense of meaninglessness and to identify the correlations between various variables. This approach offers a new perspective on psychological interventions in cataract patients.

## Methods

### Study design

This study combined cross-sectional and interventional methodologies to examine the condition of elderly cataract patients at Ningbo Eye Hospital, Wenzhou Medical University. The experimental protocol was approved by the Ethics Committee of Ningbo Eye Hospital (No. 2020-qtky-001) and adhered to the 2013 Helsinki Declaration and its subsequent amendments or comparable ethical standards.

### Participants and setting

The study was conducted from July to December 2023, utilizing random sampling to select participants from the day ward and operating room settings. The inclusion criteria specified individuals aged 60 years and older diagnosed with age-related cataracts. Exclusion criteria encompassed those with a history of ocular trauma, glaucoma, or other eye diseases, alongside those with severe mental health conditions or compromised cognitive functioning. A thorough consent process was implemented, with all participants providing written consent; caregivers provided it for those unable to consent themselves. The distribution and collection of questionnaires were coordinated to coincide with the day of admission or before the scheduled surgery. Research assistants, trained uniformly to ensure consistency, facilitated participants through the questionnaire completion process as required.

### Instruments

#### Demographics

Data on demographic characteristics, including age, gender, educational level, monthly household income, drinking habits, and types of chronic diseases, were carefully collected from participants.

#### Measurements

Participants were asked to complete the Chinese versions of the Existential Meaninglessness Anxiety Scale (EM-A) (Yanli et al., 2022), the Herth Hope Index (Zhang, 2006), and the Fear of Progression Questionnaire-Short Form (FoP-Q-SF) (Qiyun et al., 2015).

#### Existential meaninglessness anxiety scale (EM-A)

The EM-A comprises 18 items across three dimensions: incomprehension anxiety (items 1–6), purposelessness anxiety (items 7–12), and insignificance anxiety (items 13–18). It employs a 6-point Likert scale, ranging from 1 (“not anxious at all”) to 6 (“very anxious”), with higher total scores indicating greater existential anxiety. The scale’s Cronbach’s α coefficient was reported at 0.823, with a retest reliability of 0.955 in its original version (Yanli et al., 2022). The Cronbach’s α coefficient for the Chinese version was 0.97, and its retest reliability was 0.80 (Li et al., 2022).

#### Herth hope index

The Herth Hope Index, developed by Professor Herth and culturally adapted for use in China, has demonstrated strong reliability and validity, with a Cronbach’s α coefficient of 0.85 (Herth, 1991). The construct comprises three dimensions: a positive outlook on reality and the future (encompassing items 1, 2, 6, 11), engagement in proactive behaviors (items 4, 7, 10, 12), and the sustenance of strong interpersonal connections (items 3, 5, 8, 9), totaling 12 items. The assessment adopts a 4-point Likert scale methodology, wherein higher scores denote increased levels of hope. The retest reliability of the Chinese version was 0.920, and its Cronbach’s α coefficient was 0.870 (Zhang, 2006).

#### Fear of progression questionnaire-short form (FoP-Q-SF)

The FoP-Q-SF is a streamlined, unidimensional measure developed by Mehnert in Germany in 2006. Originating from the comprehensive FoP-Q, this scale version demonstrated a Cronbach’s alpha coefficient of 0.870 (Mehnert et al., 2006). Introduced to China in 2015 by a research team including Wu Qiyun, the Chinese scale adaptation reported a Cronbach’s alpha coefficient of 0.883 for the entire scale. It is segmented into two distinct dimensions: physical health and social-family well-being. The scale consists of 12 items, employing a 5-point Likert scale for responses, to be self-administered by patients. The total score ranges from 12 to 60, with higher scores indicating an increased fear of disease progression among patients (Qiyun et al., 2015).

#### Data analyses

Statistical analyses were conducted utilizing IBM SPSS Statistics version 25.0. Descriptive analysis was implemented to examine differences in demographic characteristics and associated mental health concerns. Measurement data were presented as mean (M) ± standard deviation (SD), and categorical data were represented through frequency and percentage. A threshold of *p* < 0.05 was established for statistical significance. Univariate analyses, including independent sample *t*-tests and analysis of variance (ANOVA), were employed for comparisons. A stepwise multiple linear regression was utilized in multivariate analysis to pinpoint factors associated with Existential Meaninglessness Anxiety (EM-A) in elderly patients with cataracts. Pearson’s correlation analysis was applied to explore the relationships between futile anxiety, levels of hope, and fear of disease progression. The effectiveness of the intervention was evaluated using the *t*-test (*p* < 0.05).

## Results

### Descriptive statistics in cross-sectional study

Out of 1,147 disseminated questionnaires, 1,029 were returned with valid responses, yielding a response rate of 89.71%. Statistically significant differences in the score of meaninglessness were observed across gender, age, education level, income, and type of chronic disease (*p* < 0.05). Additional demographic details of the participants are outlined in Table 1.

**Table 1:** Sample characteristics (*n* = 1,029).

Characteristics	Total (*N* = 1,029) *N* (%)	EM-A	*t*/F	*p*-value
Sex			6.163	<0.001
Woman	607 (59.0)	55.92 ± 10.13		
Man	422 (41.0)	52.10 ± 9.23		
Age			4.262	0.014
60–69	467 (45.4)	53.41 ± 9.18		
70–79	335 (32.6)	54.83 ± 10.66		
≥80	227 (22.1)	55.58 ± 10.23		
Education level			14.164	<0.001
Primary school and below	297 (28.9)	52.25 ± 9.72		
Secondary school	508 (49.4)	54.47 ± 10.01		
High school or above	224 (21.8)	56.87 ± 9.52		
Income			18.081	<0.001
0–2000	165 (29.1)	58.39 ± 10.19		
2000–4,000	485 (35.8)	54.05 ± 9.87		
≥4,000	379 (35.1)	52.97 ± 9.49		
Drink			0.916	0.401
No	397 (30.5)	54.30 ± 9.71		
Once in a while	429 (43.4)	54.02 ± 9.88		
Often	203 (26.1)	55.16 ± 10.53		
Types of chronic diseases			11.004	<0.001
One	229 (22.4)	52.02 ± 9.41		
Two	445 (43.4)	54.29 ± 9.66		
Three	355 (34.2)	55.94 ± 10.35		

### Correlation analysis

Spearman’s correlation analysis was employed to examine the relationship between the EM-A scale, the Herth Hope Level Scale, and the FoP-Q-SF. There was a negative correlation between the EM-A and hope level (*r* = −0.670, *p* < 0.01). A negative correlation was found between EMA and hope levels (*r* = −0.670, *p* < 0.01), and a positive correlation between the total scores of EMA and FoP-Q-SF (*r* = 0.890, *p* < 0.01). All dimensions of EMA negatively correlated with all dimensions of hope levels and positively correlated with all dimensions of FoP-Q-SF (Table 2).

**Table 2:** Correlation analysis.

	Incomprehension anxiety	Purposelessness anxiety	Insignificance anxiety	EM-A	Positive attitude toward the reality and future	Taking positive actions	Maintaining close relationships with others	Herth hope level scale	Physical health	Social family	FoP-Q-SF
Incomprehension anxiety	1										
Purposelessness anxiety	0.338^**^	1									
Insignificance anxiety	0.355^**^	0.429^**^	1								
EM-A	0.713^**^	0.778^**^	0.797^**^	1							
Positive attitude toward the reality and future	−0.707^**^	−0.737^**^	−0.777^**^	−0.970^**^	1						
Taking positive actions	−0.267^**^	−0.296^**^	−0.300^**^	−0.377^**^	0.365^**^	1					
Maintaining close relationship with others	−0.272^**^	−0.281^*^	−0.285^**^	−0.366^**^	0.347^**^	0.620^**^	1				
Herth hope level scale	−0.488^**^	−0.516^**^	−0.529^**^	−0.670^**^	0.669^**^	0.829^**^	0.837^**^	1			
Physical health	0.720^**^	0.755^**^	0.775^**^	0.982^**^	−0.985^**^	−0.379^**^	−0.363^**^	−0.675^**^	1		
Social family	0.440^**^	0.509^**^	0.493^**^	0.631^**^	−0.648^**^	−0.276^**^	−0.250^**^	−0.462^**^	0.648^**^	1	
FoP-Q-SF	0.640^**^	0.698^**^	0.700^**^	0.890^**^	−0.901^**^	−0.361^**^	−0.338^**^	−0.627^**^	0.910^**^	0.906^**^	1

**p<0.01; *p<0.05.

### Multiple linear stepwise regression analysis

A multiple linear stepwise regression analysis was conducted to identify the influencing factors of EMA, incorporating variables such as general information, herth hope level, and FoP-Q-SF. Age, monthly income, and education level were transformed into dummy variables for this analysis. Details are provided in Table 3. Table 4 reveals that factors such as age, education level, types of chronic diseases, herth hope level, and the FoP-Q-SF do not significantly impact anxiety levels. Furthermore, it was observed that there is a negligible effect associated with the negative influence and a notable effect with the positive influence of the FoP-Q-SF, aligning with the findings from correlation analyses.

**Table 3:** Independent variable assignment.

Variate	Assignment
Sex	Woman = 1; Man = 2
Age	60 ~ 69 = 1; 70 ~ 79 = 2; ≥80 = 3
Education level	Primary school and below = 1; Secondary school = 2; High school or above = 3
Income	0 ~ 2000 = 1; 2000 ~ 4,000 = 2; ≥4,000 = 3
Smoke	No = 1; Once in a while = 2; Often = 3

**Table 4:** Multiple linear stepwise regression analysis of meaningless anxiety scale.

Variate	*β*	SE	*Β*’	*t*	*p*-value	*R* ^2^	DR^2^	*F*	*p*-value
						0.817	0.816	91.844	<0.001
Age	0.608	0.298	0.029	2.037	0.042				
Education level	0.488	0.192	0.035	2.545	0.011				
Types of chronic diseases	0.691	0.189	0.052	3.656	<0.001				
Herth hope level	−0.333	0.032	−0.180	−10.432	<0.001				
FoP-Q-SF	1.497	0.034	0.766	44.113	<0.001				

### Descriptive statistics in intervention study

In an intervention study paralleling a cross-sectional study, an assessment of patient’s physical and mental conditions revealed a notable correlation between hope levels and EM-A, positioning these factors as crucial to patient outcomes. Building on these initial findings, a collaborative effort among ophthalmic nursing experts, head nurses, departmental nurses, and relevant doctoral professionals in ophthalmology was undertaken to develop and implement an innovative preoperative care regimen for individuals undergoing cataract surgery. This study’s nursing interventions were grounded in Orem’s Self-Care Deficit Theory, encompassing three primary strategies: total care, partial care, and health education. Given the infrequency of patients in the ophthalmology ward demonstrating complete self-care deficits, no interventions were designated for total care.

#### Partial care

Customized care plans were created for patients with limited mobility or self-care capabilities. Assigned caregivers conducted essential tasks such as patient assessments, tear duct flushes, and basic nursing actions. In the administration of eye drops, the nursing staff provided thorough and detailed explanations. Demonstrations conducted by these professionals enabled patients’ family members to comprehend the necessary procedures for eye drop care, facilitating a gradual acquisition and proficiency in preoperative care practices.

#### Health education

In the lead-up to surgery, nursing staff prioritized alleviating patients’ preoperative anxieties and enhancing their hopeful perspective on recovery, thereby reducing feelings of existential meaninglessness. The nursing staff consistently held educational sessions addressing essential preoperative care knowledge, detailing the intraoperative and postoperative phases and the anticipated recovery outcomes. Patients were advised to seek assistance from the nursing staff for any concerns encountered during the self-care regimen.

This intervention study involved 317 patients, and for the cross-sectional study, 317 individuals were randomly selected for the control group. Comparative analysis before and after the intervention (presented in Table 5) demonstrated a significant reduction in both the mean and total scores across all dimensions of meaninglessness (*p* < 0.05).

**Table 5:** Before and after the intervention of analysis of meaningless anxiety scale.

Project	Incomprehension anxiety	Purposelessness anxiety	Insignificance anxiety	EM-A
Pre-intervention	17.91 ± 2.94	17.44 ± 3.19	16.73 ± 3.07	52.08 ± 2.32
Post-intervention	16.79 ± 3.08	16.56 ± 3.74	16.09 ± 3.24	49.44 ± 6.34
*t*	4.685	3.198	2.541	6.970
*p*	<0.001	0.001	0.011	<0.001

## Discussion

### Current state of existential meaninglessness in patients with senile cataracts

Life’s unpredictability, marked by health crises, chronic diseases, family disruptions, and natural disasters, can profoundly unsettle individuals, sparking intense concerns over life’s meaning (Yanli et al., 2022). This existential distress not only impairs emotional and psychological well-being but can also accelerate disease progression (Wang and Feng, 2022). This effect is especially pronounced in individuals facing chronic illnesses (Mahmood et al., 2021), where ongoing health challenges and the impact of external, uncontrollable factors deepen the existential meaninglessness (Zhou et al., 2020; King and Hicks, 2021). Cataracts, as a chronic eye condition, exemplify the critical need to assess and address the psychological aspects of physical health ailments (Hou et al., 2021). This cross-sectional survey engaged 1,029 elderly patients with senile cataracts from Ningbo City, Zhejiang province, uncovering a significantly high score of the EM-A. The study’s findings indicate that elderly individuals with chronic conditions report an EM-A score of 54.35 ± 9.95, which exceeds the critical threshold compared to the midpoint of the scale’s total score. Contrary to similar studies, this cohort of cataract patients displayed lower EM-A scores (Xue et al., 2023). This difference may be attributed to the gradual progression of cataracts, which minimally affects patients’ quality of life and leads to limited emotional fluctuations (Moreau and King, 2012). Alternatively, it could be due to patients in the early stages of cataract development and maintaining relatively good visual acuity (Ahsan et al., 2021).

Our analysis identified a significant correlation between gender and existential meaninglessness. Notably, women scored higher than men, possibly due to a higher propensity for mood fluctuations as the cataract disease progresses (Kohn et al., 2019). Concerning age, the feeling of meaninglessness intensifies with advancing years, likely due to increased loneliness among the elderly, exacerbated by the progression of cataracts, which leads to blurred vision and reduced visual acuity (Dong et al., 2023). Interestingly, an inverse relationship was found with education levels; individuals with higher educational attainment exhibited more pronounced feelings of meaninglessness. This result could be because those with a greater educational background pay more attention to health concerns, have a better understanding of diseases, and experience increased anxiety about disease progression (Letta et al., 2023). Additionally, the study highlighted a trend where individuals with higher income levels scored lower on the meaninglessness scale, suggesting that better economic conditions might mitigate emotional distress (Bøe et al., 2020). The consumption of occasional alcoholic beverages also seemed to positively influence patients’ psychological states, potentially due to the mild stimulating effects of moderate alcohol intake (Klonowski, 2007). Moreover, many diseases were associated with increased feelings of meaninglessness (Chen et al., 2020). This relationship may stem from the complex emotional responses of patients who are not only dealing with a primary ailment but are also managing multiple health issues (Morita et al., 2007).

### Relationship of the anxiety measure of existential meaninglessness, hope level, and fear of progression among senile cataract patients

The early stages of cataract development typically do not cause significant discomfort, complicating early detection (Nakazawa et al., 2020). By the time the condition is recognized, many patients have advanced to the swelling and maturation phases. Some may even mistakenly attribute symptoms of presbyopia, a natural part of aging, to the aging process of their eyes (Nakazawa et al., 2020). These uncontrollable external factors accelerate disease progression, causing anxiety among patients (Forbes et al., 2020). As visual impairment worsens, patients may also begin to feel a deep sense of hopelessness toward life (Meyer-Rochow et al., 2015). Through Pearson correlation analysis, we discovered a link between the sense of meaninglessness and levels of hope and fear. Specifically, variables such as Incomprehension anxiety, Purposelessness anxiety, and EM-A demonstrated negative correlations with the Herth Hope Level Scale (*r* = −0.488, −0.516, −0.529, −0.670, *p* < 0.01), indicating that decreased levels of hope are associated with an intensified sense of meaninglessness. This finding aligns with research indicating that lower hope levels are linked to a higher likelihood of anxiety and depression (Zeng et al., 2021) and are correlated with a diminished quality of life (Alshraifeen et al., 2020). These findings mirror the patterns observed in our study.

Scholars have argued that individuals with chronic illnesses often experience increased fears of disease progression (Wang et al., 2022) and that moderate to severe anxiety about disease advancement can exacerbate anxiety levels, thereby reducing self-efficacy (Folkerts et al., 2022). Extensive literature has highlighted the widespread occurrence of generalized and persistent anxiety within the general populace (Crocq, 2017), which may escalate to mental and psychological disorders, underscoring the need for intervention (Mohlman, 2020). In our study, Incomprehension anxiety, Purposelessness anxiety, and EM-A showed positive correlations with the FoP-Q-SF (*r* = 0.488, 0.516, 0.529, 0.670, *p* < 0.01), suggesting that increased fears of disease progression exacerbate feelings of meaninglessness in patients, thereby affirming the detrimental impact of such fears on mental health (Zhu et al., 2015).

Upon converting general data into dummy variables, our multiple linear regression analysis indicated that age, education level, and type of chronic illness play significant roles in contributing to the sense of EM-A in elderly patients with senile cataracts. This finding diverges from other research, possibly due to differences in sample selection, and points to the need for further studies encompassing a wider array of regions. Notably, older age was associated with a decreased presence of anxiety in elderly individuals with senile cataracts, which might be attributed to external factors such as physical health, activity levels, and family support (Alsubaie et al., 2020). Conversely, higher education levels and lower monthly incomes were correlated with less severe anxiety among these patients, likely influenced by their awareness of the disease and their financial situation (Gase et al., 2017; Knifton and Inglis, 2020). Additionally, the frequency of alcohol consumption was observed to have varying impacts on physical and psychological well-being (Hafford-Letchfield et al., 2020). An increased frequency of interventions leads to significantly greater physical stress and fatigue, subsequently elevating the sense of anxiety related to feelings of meaninglessness among elderly patients with senile cataracts (Song et al., 2022).

Levels of hope and fear are identified as significant influencers in the perception of meaninglessness among elderly patients with senile cataracts. Specifically, hope exerts a negative influence, acting as a mitigating factor, whereas fear contributes positively, amplifying the sense of meaninglessness. A higher level of hope was associated with improved psychological states, contributing to reduced anxiety about life and the disease (Mardhiyah et al., 2020). Conversely, lower levels of fear regarding disease progression were connected with decreased anxiety (Licht et al., 2021). Therefore, in the clinical treatment and early intervention stages, it may be advantageous to proactively manage these factors to address the psychological well-being of patients. Emphasis should be placed on the mental health of patients, enhancing their social adaptability and life quality, and fostering positive societal development.

### Effectiveness of preoperative care based on Orem’s theory

Nursing plays a vital role in patients’ holistic medical care journey (Skuban-Eiseler et al., 2023), with preoperative care before cataract surgery emerging as a critical area for innovation through the application of Orem’s theory. Previous research, including a cross-sectional study, has highlighted the correlation between levels of hope, fear, and a sense of meaninglessness, pinpointing these as key factors influencing patient outcomes. In response, we developed a novel set of preoperative care practices for cataract patients rooted in Orem’s theory. Engaging 317 patients, our study showcased significant benefits. Specifically, the group receiving the novel care approach showed notably lower scores in Incomprehension anxiety, Purposelessness anxiety, Insignificance anxiety, and EM-A compared to the control group, with all differences achieving statistical significance (all *p* < 0.05). The findings demonstrated reductions in the mean scores across each dimension, from 17.91, 17.44, 16.73, and 52.08 to 16.79, 16.56, 16.09, and 49.44, respectively. Overall, the experimental group experienced a slight decline in mean scores across dimensions and the entire scale. This trend could be attributed to the specific patient management practices associated with day surgery, characterized by limited time for preoperative preparation and entry into surgery with an incomplete understanding of health education and disease knowledge (Freundlich et al., 2020; Kym et al., 2023). Nevertheless, the application of preoperative care guided by Orem’s theory has proven effective in enhancing patient comprehension of preoperative care, alleviating preoperative fears, and mitigating apprehension about the disease. Research supports that interventions based on Orem’s theory markedly improve life quality, self-care, and self-efficacy and significantly reduce anxiety and depression (Khademian et al., 2020; Tok Yildiz and Kaşikçi, 2020; Nasiri et al., 2023). This study, integrating relevant nursing interventions, further corroborates the clinical utility of the theory, significantly enhancing the quality of eye care and enriching patients’ medical experiences, thereby bolstering public confidence in healthcare services.

### Limitations

While this study provides valuable insights, it’s essential to recognize its limitations to refine future research. The cross-sectional design of our study limits our ability to infer causality between observed variables. Future investigations could adopt longitudinal methods to elucidate the temporal relationships and causal mechanisms underlying existential meaninglessness among cataract patients. Our sample predominantly consisted of older adults, introducing the possibility of sociodemographic bias. Including a wider range of ages in the study population would offer a broader perspective on existential meaninglessness across various life stages. Moreover, the focus on a specific geographic area and population might constrain the applicability of our findings to other cultural and socioeconomic contexts. Conducting similar studies in diverse settings would enhance the external validity and global relevance of our results.

## Conclusion

In summary, our study, which utilized a preoperative care model grounded in Orem’s theory, has emphatically validated the effectiveness of this approach in improving the physical and mental well-being of patients. It facilitates a swifter recovery process and enhances the quality of life for patients. This positive impact of nursing care extends beyond the immediate benefits to patients and their families; it also significantly mitigates the societal medical burden and contributes to the harmonious development of the community. Our research provides a strong theoretical foundation for future nursing practices and offers valuable insights into the continuous exploration and enhancement of preoperative care models for cataracts.

## Data availability statement

The original contributions presented in the study are included in the article/supplementary material, further inquiries can be directed to the corresponding author.

## Ethics statement

The studies involving humans were approved by all procedures performed in studies involving human participants were approved with the ethical standards of the Ethics Committee of Ningbo Eye Hospital. The studies were conducted in accordance with the local legislation and institutional requirements. The participants provided their written informed consent to participate in this study. Written informed consent was obtained from the individual(s) for the publication of any potentially identifiable images or data included in this article.

## Author contributions

YZ: Conceptualization, Writing – original draft, Writing – review & editing. YC: Investigation, Writing – original draft, Writing – review & editing. YL: Methodology, Writing – original draft, Writing – review & editing. MS: Methodology, Writing – original draft, Writing – review & editing. AC: Data curation, Writing – original draft, Writing – review & editing.
